# An 800-year record of benthic foraminifer images and 2D morphometrics from the Santa Barbara Basin

**DOI:** 10.1038/s41597-024-02934-9

**Published:** 2024-01-30

**Authors:** Sara S. Kahanamoku, Maya Samuels-Fair, Sarah M. Kamel, Da’shaun Stewart, Bryan Wu, Leah X. Kahn, Max Titcomb, Yingyan Alyssa Mei, R. Cheyenne Bridge, Yuerong Sophie Li, Carolina Sinco, Julissa Moreno, Josef T. Epino, Gerson Gonzalez-Marin, Chloe Latt, Heather Fergus, Ivo A. P. Duijnstee, Seth Finnegan

**Affiliations:** 1 Department of Integrative Biology and Museum of Paleontology, University of California at Berkeley, Berkeley, CA 94720 USA; 2 Department of Psychology, University of California at Berkeley, Berkeley, CA 94720 USA; 3 Department of Molecular and Cell Biology, University of California at Berkeley, Berkeley, CA 94720 USA; 4 Department of Environmental Science, Policy, and Management, University of California at Berkeley, Berkeley, CA 94720 USA; 5 Department of Electrical Engineering and Computer Sciences, University of California at Berkeley, Berkeley, CA 94720 USA; 6 Department of Earth and Planetary Science, University of California at Berkeley, Berkeley, CA 94720 USA; 7 Scripps Institution of Oceanography, University of California at San Diego, La Jolla, CA 92093 USA; 8 College of Chemistry, University of California at Berkeley, Berkeley, CA 94720 USA; 9 Department of Sociology, University of California at Berkeley, Berkeley, CA 94720 USA; 10 Present Address: School of Ocean and Earth Sciences and Technology, University of Hawai’i at Mānoa, Honolulu, HI 96822 USA

**Keywords:** Palaeoecology, Marine biology, Climate-change ecology

## Abstract

The Santa Barbara Basin is an extraordinary archive of environmental and ecological change, where varved sediments preserve microfossils that provide an annual to decadal record of the dynamics of surrounding ecosystems. Of the microfossils preserved in these sediments, benthic foraminifera are the most abundant seafloor-dwelling organisms. While they have been extensively utilized for geochemical and paleoceanographic work, studies of their morphology are lacking. Here we use a high-throughput imaging method (*AutoMorph*) designed to extract 2D data from photographic images of fossils to produce a large image and 2D shape dataset of recent benthic foraminifera from two core records sampled from the center of the Santa Barbara Basin that span an ~800-year-long interval during the Common Era (1249–2008 CE). Information on more than 36,000 objects is included, of which more than 22,000 are complete or partially-damaged benthic foraminifera. The dataset also includes other biogenic microfossils including ostracods, pteropods, diatoms, radiolarians, fish teeth, and shark dermal denticles. We describe our sample preparation, imaging, and identification techniques, and outline potential data uses.

## Background & Summary

Morphological data are the primary phenotypic data preserved in the fossil record. Yet until recently, information on morphological variation within fossil assemblages was limited by the laborious nature of manual morphological data collection. Recent advancements in data collection and processing techniques, such as the adoption of rapid two- and three-dimensional imaging techniques, have greatly accelerated the pace with which researchers can gather large morphological datasets^1^. However, even with the aid of technological advancements, the collection of morphological information from individual specimens at the population, community, or assemblage scale remains relatively rare, as assessment of trends at these levels requires large amounts of data that remains time-intensive to collect. To address this gap, paleontologists have developed high-throughput approaches for extracting 2D and 3D shape information from photographic images of entire populations or assemblages. One of these approaches, *AutoMorph*^2^, which is used primarily for the creation of large microfossil datasets, has led to an explosion of big data in micropaleontology, as datasets generated with this method contain thousands to tens of thousands of individuals^3,4^. These data have subsequently driven major scientific discoveries, including the identification of a previously unknown potential extinction event in sharks^5^ and a morphological diversification event in fishes^6,7^.

While big datasets have been generated for a number of marine microfossil groups, benthic foraminifera have largely been left out of the paleo big data revolution. No major morphological datasets have been generated for benthic foraminifera, and the vast majority of studies employ manual counting or description of morphotypes^8,9^, labor-intensive morphometry techniques^10–12^, or rely on species- or genus-level exemplar specimens to describe trends within benthic foraminifer assemblages through time^13^. These manual techniques are not only time-consuming, but are also difficult to replicate without specialized knowledge and access to the physical samples used in a given study.

Benthic foraminifera are a useful focus group for the development of large, individual-level morphological datasets, as these unicellular protists have calcium carbonate tests (i.e., shells) that are distributed throughout the benthos of the modern global ocean, and are cosmopolitan within marine sediments, living anywhere from littoral to deep-water environments^14^. The abundant fossil record of benthic foraminifera spans back to the early Cambrian^15,16^, and as a result has been extensively utilized to examine eco-environmental trends through time. These include past environmental conditions, assessed using species distribution data and shell chemistry, among other proxies^14,17–19^, as well as and ecological and evolutionary trends, assessed using genus- and species-level diversity data, classification of ecophenotypes, and quantitative assessments of lineage diversification and extinction^20–22^.

**Fig. 1:**
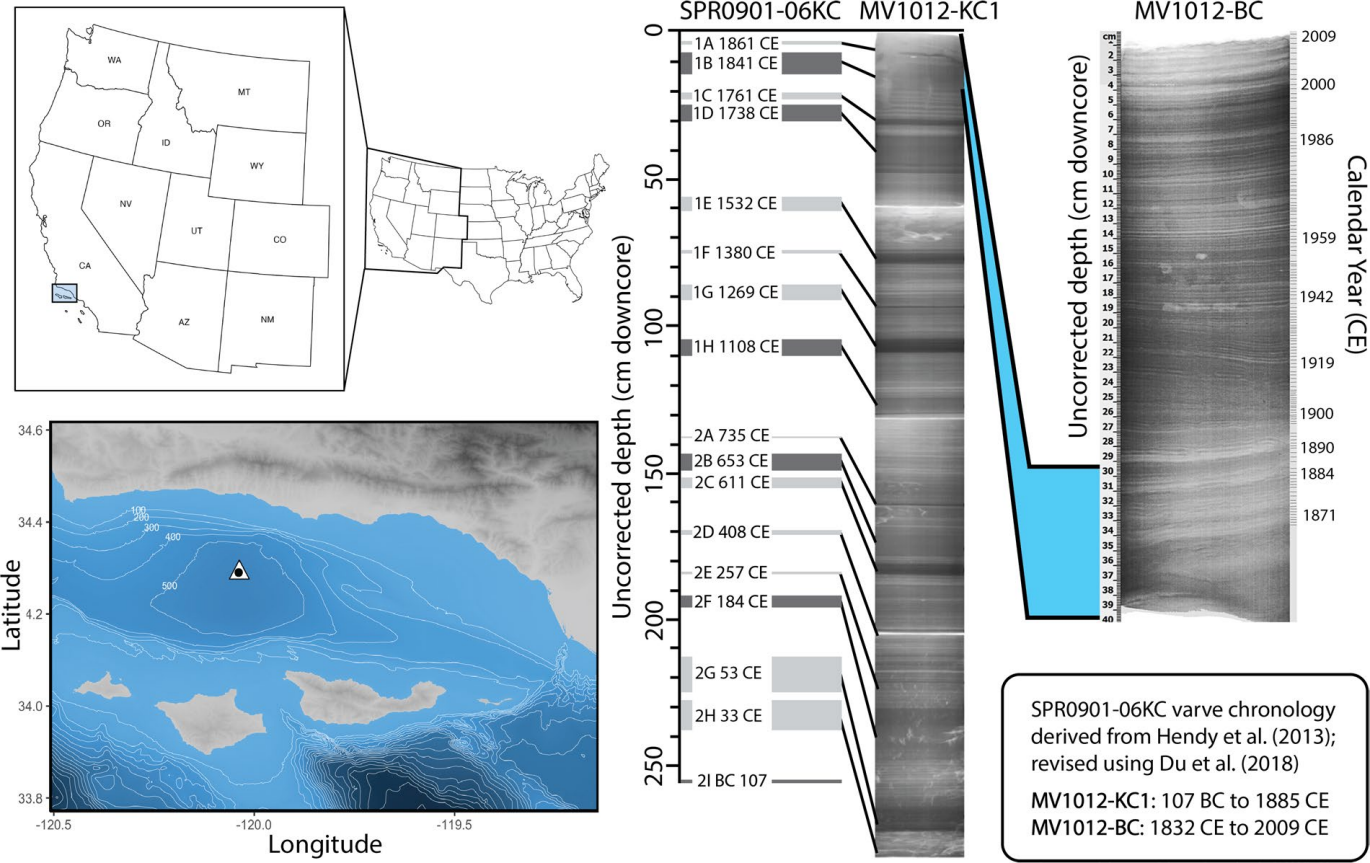
Regional setting and core materials. (Left) The sampling location for kasten and box cores, site MV1012-ST46.9, (34°17.228′ N, 120°02.135′ W), is denoted by a white triangle. The sampling location was chosen as a reoccupation of Ocean Drilling Program site 893 (34°17.25′ N, 120° 2.16′ W, 577 m water depth), denoted by a black circle. Contour lines indicate seafloor depth (m). (Right) Core chronology for box core MV1012-BC-1 and kasten core MV1012-KC1. Core images modified from Jones 2016 and Brandon *et al*.^34^.

In some highly resolved systems, benthic foraminifera are preserved at seasonal to decadal resolution. One such extraordinary site is the Santa Barbara Basin (SBB), where persistent hypoxia within the center of the basin preserves seasonal varve couplets. The dysoxic bottom waters of the SBB result in part from its bathymetry, where its relatively high eastern (230 m) and western (475 m) sill depths (Fig. 1) restrict intermediate water movement. This hypoxia is further intensified by overlying surface productivity in the Santa Barbara Channel^23^, which typically serves to exclude large bioturbators from the center of the basin^24–26^. Basin-enhanced hypoxia^27^, coupled with high seasonal sedimentation rates into the basin (on the order of 140 cm ky^−1^), aid in the preservation of millimeter-scale couplet pairs for which the basin is famous^28^. To date, these varved sediments have been extensively used to generate long-term records of climate variability, making the SBB one of the most well-studied marine systems in the world. However, considerably less focus has been paid to developing high-resolution morphological records from the basin’s fossiliferous material, leaving a considerable data gap that stands to be addressed with automated approaches.

While there is a history of (semi-) automated approaches being used on benthic foraminifer taxa to extract information such as size^29^, 2D shape^8,30^, calcite thickness^31^, life history variation^11,32^, and biovolume through ontogeny^13,33^, these datasets are rarely publicly available in their raw forms. This combined lack of data on individual-level trends and a lack of accessibility for those data that do exist have limited the ability of additional studies to build on previous results and describe trends at population and community scales. As biologists increasingly strive to elucidate the impacts of climate change on marine life at all scales, large, individual-level datasets that span across historic periods of environmental change are critical both for building ecosystem baselines that place modern change into context and for leveraging the predictive power of the fossil record to understand the range of biological responses expected under projected climate scenarios.

Existing workflows can be used to address benthic foraminifer data gaps and begin to build large, open-access datasets that collect information on the individual-level intraspecific morphological trait distributions needed to reconstruct community ecological characteristics. Here we provide an image library of individual benthic foraminifera and high-resolution 2D assemblage images, individual images, coordinate data, and morphometric measurements from Santa Barbara Basin (SBB) sediment core samples. Images of 27,508 complete and damaged benthic foraminifera are provided along with 2D morphometric data. Images and shape data for an additional ~1,000 objects are also provided, encompassing general categories including planktonic foraminifera, ostracods, pteropods, diatoms, radiolarians, fish teeth and skeletal structures, shark dermal denticles, and foraminiferal test fragments (see Methods for further information). Benthic foraminifera are identified to species, and classifications of reproductive mode (i.e, sexually vs. asexually produced offspring) are included for four biserial species with significant and visible dimorphism that allows for examination of life history trends among reproductive morphotypes.

## Methods

### Core sampling and chronology development

As part of previous studies^34,35^, a kasten core and a box core from the center of the SBB (Southern California) were collected in 2010 at station MV1012-ST46.9 (34°17.228′N, 120°02.135′W) at approximately 580 m water depth (Fig. 1). This station was chosen as a re-occupation of Ocean Drilling Program Site 893^36^ and was designated as Station 46.9 following the station naming convention of the California Cooperative Oceanic Fisheries Investigations (CalCOFI)^37,38^. 2-cm vertical core slices from each subcore were X-radiographed and scanned at 1-mm intervals in a linear, non-rotational scan^34,35^. Composite X-radiographs were used with color photographs to develop a high-resolution chronology for each core (Fig. S1). The age model for the kasten core MV1012-KC1 was adapted from Hendy *et al*.^39^ and Schimmelmann *et al*.^40^; dates assigned to each sample were the average of the dates of the upper and lower surfaces of the sample transverse section. Kasten core MV1012-KC1 spans from 107 BC to 1885 CE (Fig. 1); however, we utilized a subset from 1249 CE to 1841 CE to develop the present dataset (Table 1). Box core MV1012-BC1 was sufficiently shallow to use traditional varve chronology for couplet dating; a regression model was used to assign dates to the sediment stratigraphy prior to 1871, thus extending the chronology to 1834 CE^34^.

**Table 1:** Sample ages and split fractions.

Core Type	Sample	Box Core Depth (cm)	Kasten Core Depth (cm)	Calendar Year (CE)	Assemblage Type	Split Size
Box	MV1012-BC-2	0.5		2007.9	All	1
Box	MV1012-BC-3	1		2006.7	All	1
Box	MV1012-BC-4	1.5		2005.6	All	1
Box	MV1012-BC-5	2		2004.4	All	1
Box	MV1012-BC-6	2.5		2003.3	All	1
Box	MV1012-BC-7	3		2002.1	All	1
Box	MV1012-BC-8	3.5		2001	All	1
Box	MV1012-BC-9-10	4		1998.4	All	1
Box	MV1012-BC-11	5		1995.8	All	1
Box	MV1012-BC-12	5.5		1994	All	1
Box	MV1012-BC-14	6.5		1990.5	All	1
Box	MV1012-BC-15	7		1988.8	All	1
Box	MV1012-BC-16	7.5		1987	All	1
Box	MV1012-BC-17	8		1984.8	All	1
Box	MV1012-BC-19	9		1980.5	All	1
Box	MV1012-BC-20	9.5		1978.4	All	1
Box	MV1012-BC-22	10.5		1974.1	All	1
Box	MV1012-BC-25	12		1967.6	All	1
Box	MV1012-BC-26	12.5		1965.5	All	1
Box	MV1012-BC-27	13		1963.3	All	1
Box	MV1012-BC-32	15.5		1952.6	All	1
Box	MV1012-BC-36	17.5		1944.1	All	1
Box	MV1012-BC-41	20		1931.8	All	1
Box	MV1012-BC-42	20.5		1929.2	All	1
Box	MV1012-BC-48	23.5		1913.6	All	1
Box	MV1012-BC-53	26		1900	All	1
Box	MV1012-BC-57	28		1890.9	All	1
Box	MV1012-BC-70	34.5		1862.3	All	1
Kasten	KC1-1-25		12	1841	Biserial only	1/32
Box	MV1012-BC-82	40.5		1836.3	All	1
Box	MV1012-BC-83	41		1834.2	All	1
Kasten	KC1-1-52		25.5	1820	Biserial only	1/32
Kasten	KC1-1-64		31.5	1769	Biserial only	1/16
Kasten	KC1-1-103		51	1712	Biserial only	1/128
Kasten	KC1-1-117		58	1666	Biserial only	1/16
Kasten	KC1-1-124		61.5	1643	Biserial only	1/32
Kasten	KC1-2-9		66.5	1610	Biserial only	1/64
Kasten	KC1-2-28		76	1548	Biserial only	1/32
Kasten	KC1-2-49		86.5	1478	Biserial only	1/32
Kasten	KC1-2-59		91.5	1429	Biserial only	1/32
Kasten	KC1-2-64		94	1405	Biserial only	1/16
Kasten	KC1-2-68		96	1385	Biserial only	1/128
Kasten	KC1-2-80		102	1325	Biserial only	1/32
Kasten	KC1-2-87		105.5	1289	Biserial only	1/16
Kasten	KC1-2-103		113.5	1249	Biserial only	1/32

### Sample preparation

Prior to the present study, subcore cross-sections were cut transversely at every 0.5 cm to create transverse sections of 97.5cm^3^, and these were stored at −80 °C prior to further processing. Near-instantaneous event layers were combined with chronologically-correlated transverse sections to create larger samples. These sections were dried overnight at 50 °C, washed in distilled water, and wet-sieved over a 104- and 63-μm mesh. Two previous studies, Jones and Checkley^35^ and Brandon *et al*.^34^ picked the >104 μm fractions for fish otoliths and plastic particles, respectively. To generate the present dataset, we picked samples from the 63–104 and >104 μm fractions for benthic foraminifera. These fractions were combined to create a single >63 μm fraction for all cores. Kasten core samples were dry split using a sediment splitter to achieve aliquots of approximately equivalent sample volumes for subsample picking, while box core samples were processed in their entirety. Kasten core samples were picked exclusively for biserial benthic foraminifera, while box core samples were picked for all benthic foraminifer individuals present within a given sample. Split fractions and community data types (biserial only, or representative of the full benthic foraminifer community) are reported in Table 1.

### Imaging

We imaged all benthic foraminifera picked from entire samples or representative split fractions. Benthic foraminifera were manually picked from each sample or split under a Leica EZ4 dissecting microscope at 16x magnification, and were arranged for imaging on matte black coated brass plates. Arranging ensured that individual foraminifera and other objects were not touching, a critical step for simplified post-processing using high-throughput imaging techniques^2^. On the few occasions that all sample material did not fit within the boundaries of a single plate, multiple plates were imaged and named accordingly (e.g., MV1012-BC-40_1, MV1012-BC-40_2, etc.). Arranged samples were imaged in bulk using a Keyence VHX-7000 digital imaging microscope at 150x magnification, and the same lighting settings were used across samples to improve comparability. Each sample took between 30 minutes to 4 hours to image; runtime was dependent on the number of individuals present in each given sample.

### AutoMorph (automated morphometric post-processing)

**Fig. 2:**
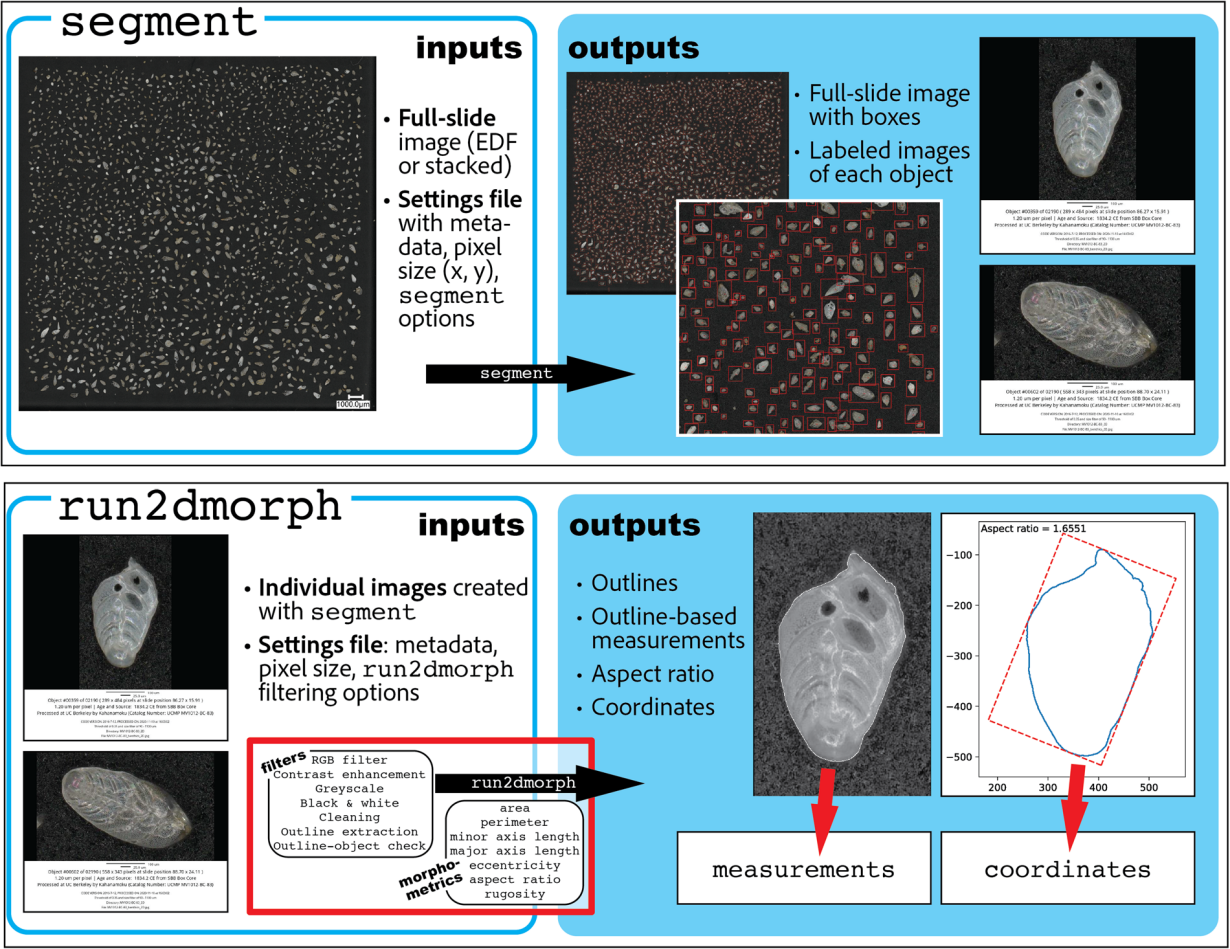
High-throughput imaging and AutoMorph image processing protocols. *AutoMorph* is an open-source software suite used for high-throughput image processing and automated morphometric measurements. For this study, two *AutoMorph* modules were used: segment (top panel) and run2dmorph (bottom panel). Segment takes as an input a full slide image and a settings file with metadata (sample name, age, location of collection, catalog number, etc.), size information (typically expressed as pixel size, e.g. microns per pixel), and settings flags. Segment outputs include a full-slide image with boxed and numbered individual objects, which correspond to individual images of objects, which are labeled with metadata as well as a scale bar. Run2dmorph takes as input the individual images created with segment (for this study, EDF images) as well as a settings file with measurement and filtering flags. Run2morph processes individual images through filters to create outlines, and uses outlines to generate outline-based measurements of area, perimeter, major and minor axis length, eccentricity, aspect ratio, and rugosity. Outlines and aspect ratios are output as images for visual checks, and measurements and outline coordinates are output as CSV files.

Bulk images were processed with the *AutoMorph* software package (v. 2017-06)^2^, an open-access bioinformatics pipeline designed to segment individual objects from light microscope and camera images and extract 2D and 3D shape information. The *AutoMorph* protocol contains four modules for 2D and 3D image processing: *segment, focus, run2Dmorph*, and *run3Dmorph*. For this study, the *segment* module was used to identify all unique objects in a 2D extended-depth-of-focus (EDF) bulk image, extract these objects and label them with sample metadata, and save these slices in unique directories. Because the bulk images used for this study were already compiled into EDF images, the *focus* module, which is designed to compile z-slices into EDF images, was not needed. Once images were segmented, we used the *run2dmorph* module to extract shape coordinates and basic measurements in 2D and create images of 2D shape extraction for visual quality control (Fig. 2). The *AutoMorph* software package and documentation is freely available on GitHub and can be accessed at https://github.com/HullLab/AutoMorph. The software suite and resultant datasets are described in detail in several publications^2–4,41^. *AutoMorph* is adapted to run on local computers and supercomputer clusters; for this study, a laptop computer with a 2.6 GHz Quad-Core Intel i7 processor was sufficient to process all samples. *AutoMorph* processing is relatively quick; runtime was on the order of minutes for each sample processed with both *segment* (per bulk image) and *run2dmorph* (per thousand individual images).

### Image identification

Individual images produced by the *segment* module were used to identify all unique objects (Table 2) using a custom-made application for image viewing and the assignment of general object information to images, including the certainty of object classification. This application, called classifier, is a modified version of *classify-specify* (https://github.com/HullLab/Classify-Specify) designed for use on unix systems. The classifier application and documentation can be accessed at https://github.com/GregDMeyer/classifier. For samples with multiple bulk images, object numbers for each image following the first were modified, typically by adding an additional number to the beginning (e.g., obj. 00001 of the second bulk image becomes obj. 10001; for the third, obj. 20001, etc.) to avoid overlapping numbers. These allowed for smooth classification using the classifier application.

**Table 2:** Major object classification categories and brief definitions.

Classification Category	Definition
Junk	Fibers, imaging plate background, rocks, inorganic crystalline structures, and poorly-extracted partial images of benthic foraminifera
Planktic	Whole, damaged, or partial planktonic foraminifera, including shell wall fragments identifiable as planktonic
Fragment	Fragments of benthic foraminifera unidentifiable to species or too incomplete to serve as a morphological specimen
Gastropod	Microgastropods or any gastropod fragments; does not include pteropods
Fish Tooth	Whole, damaged, or partial fish teeth
Dermal Denticle	Shark dermal denticles or placoid scales
Diatom	Whole, partial, or damaged diatom frustule
Spicule	Sponge spicules, typically microscleres
Echinoid	Whole or fragmented echinoid spines
Pteropod	Whole, damaged, or partial pteropod shells
Radiolarian	Whole, damaged, or partial radiolarian tests
Ostracod	Whole, damaged, or partial ostracod valves or carapaces
Touching	Segmented images of multiple objects, typically objects with overlapping outlines
Bivalve	Larval bivalve shells, small whole bivalve specimens, or identifiable shell fragments

### Preparation for machine learning workflows

A subset of 10,827 images was formatted for use with Tensorflow workflows, a commonly-used open-source platform for machine learning^42^. We used Tensorflow v. 2.12.0 (2022) to develop the models reported on in this analysis. Images were stripped of AutoMorph-generated metadata labels and resized to 224 × 224 pixels for standardization. This machine learning image dataset includes two label types: one label for species identity (originally encoded numerically, from 0–54, with the encoding 0 denoting objects other than benthic foraminifera) and a second boolean label for the fragmentation state of the shell (1 denoting broken, 0 denoting intact). This dataset has been split into subsets for training (80%), validation (10%), and testing (10%), and data augmentation has been omitted in the presentation of the dataset. The Tensorflow dataset is also provided as a zip file containing the image dataset and its corresponding Tensorflow dataset descriptor (see Data Records).

**Fig. 3:**
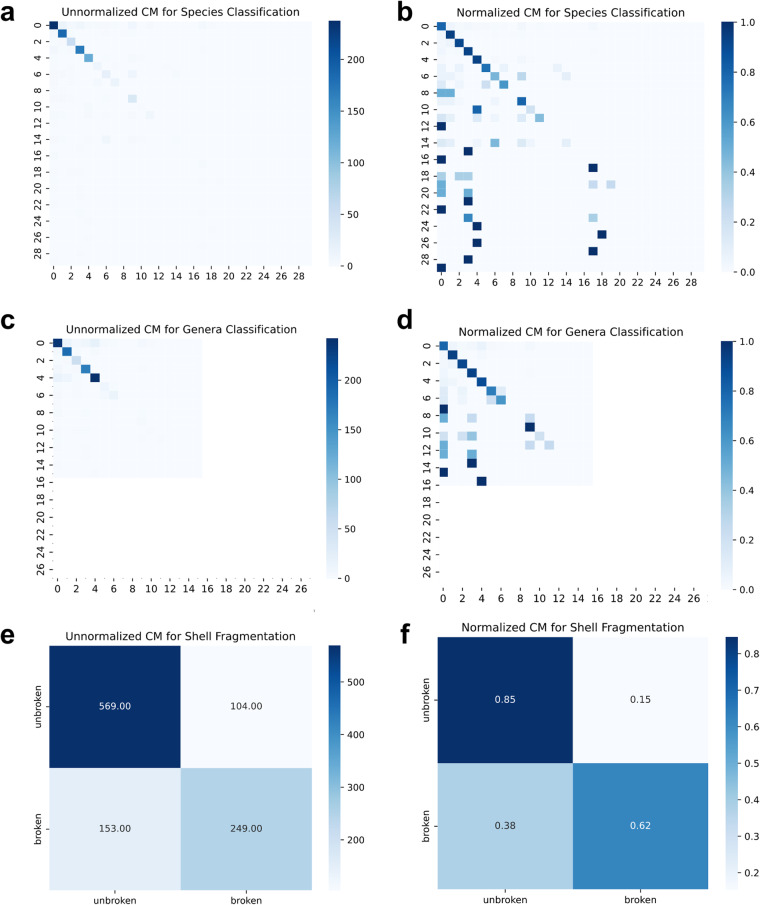
Confusion matrices of a resnet-50 transfer learning classifier. Note that this model is simultaneously classifying species and specimen fragmentation state. Panels a and b show species classification confusion matrices while panels c and d show genera classification confusion matrices, where a and c are unnormalized and b and d are normalized. Normalization scales values across each ground-truth label (i.e., row) such that they sum to 1; thus, the color saturation represents the fraction of that true label that was classified for each predicted label (where greater saturation indicates more images in the category). Confusion matrices for species classification shows that only extremely rare species are heavily misclassified (typically as a non-foram object). Panels e and f show unnormalized and normalized confusion matrices for fragmentation state, respectively. In this use case, the classifier tends to misclassify the fragmentation state for fragmented shells, but not for complete shells. This standard, unoptimized transfer learning classification approach has validation accuracies of 80.6% (species), 85.6% (genus), and 76.1% (fragmentation state).

We applied a resnet-50 transfer learning classifier to simultaneously classify species identity and specimen fragmentation state. We found that this classification approach has a validation accuracy of 80.6% for species and 85.6% for genera (Fig. 3). This demonstrates that even standard, unoptimized transfer learning model architectures can be successful for automated identification of benthic foraminifera, particularly for usage applications where common species are considered (see Usage Notes).

## Data Records

**Fig. 4:**
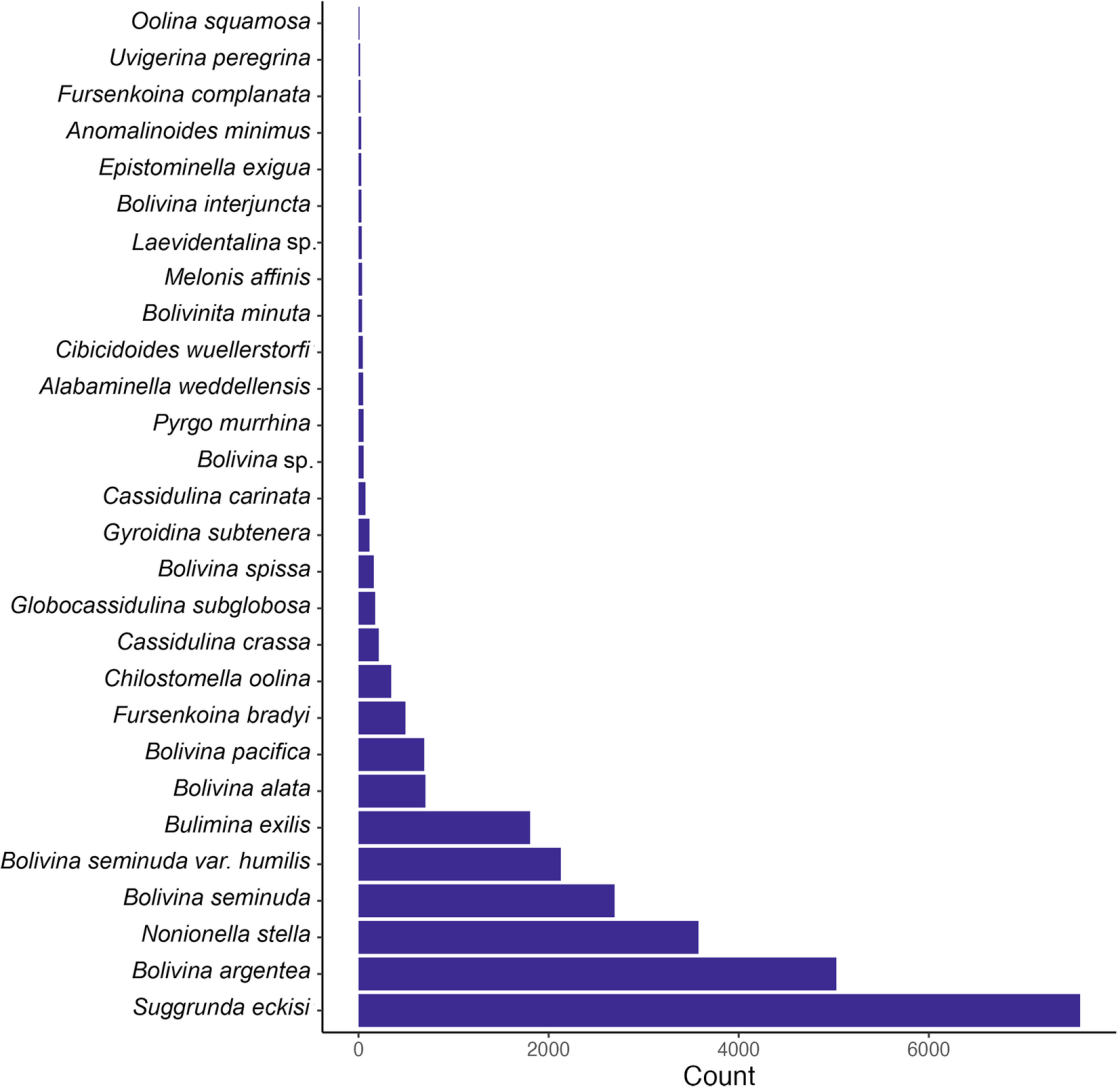
Histogram of species abundances within the dataset. Counts are shown for species with more than 10 occurrences within the entire data record (28 of the 77 total unique species represented in the data). Six common species (*Suggrunda eckisi*, *Bolivina argentea*, *Nonionella stella*, *Bolivina seminuda*, *Bolivina seminuda var. humilis*, and *Bulimina exilis*) make up the majority of the dataset.

**Fig. 5:**
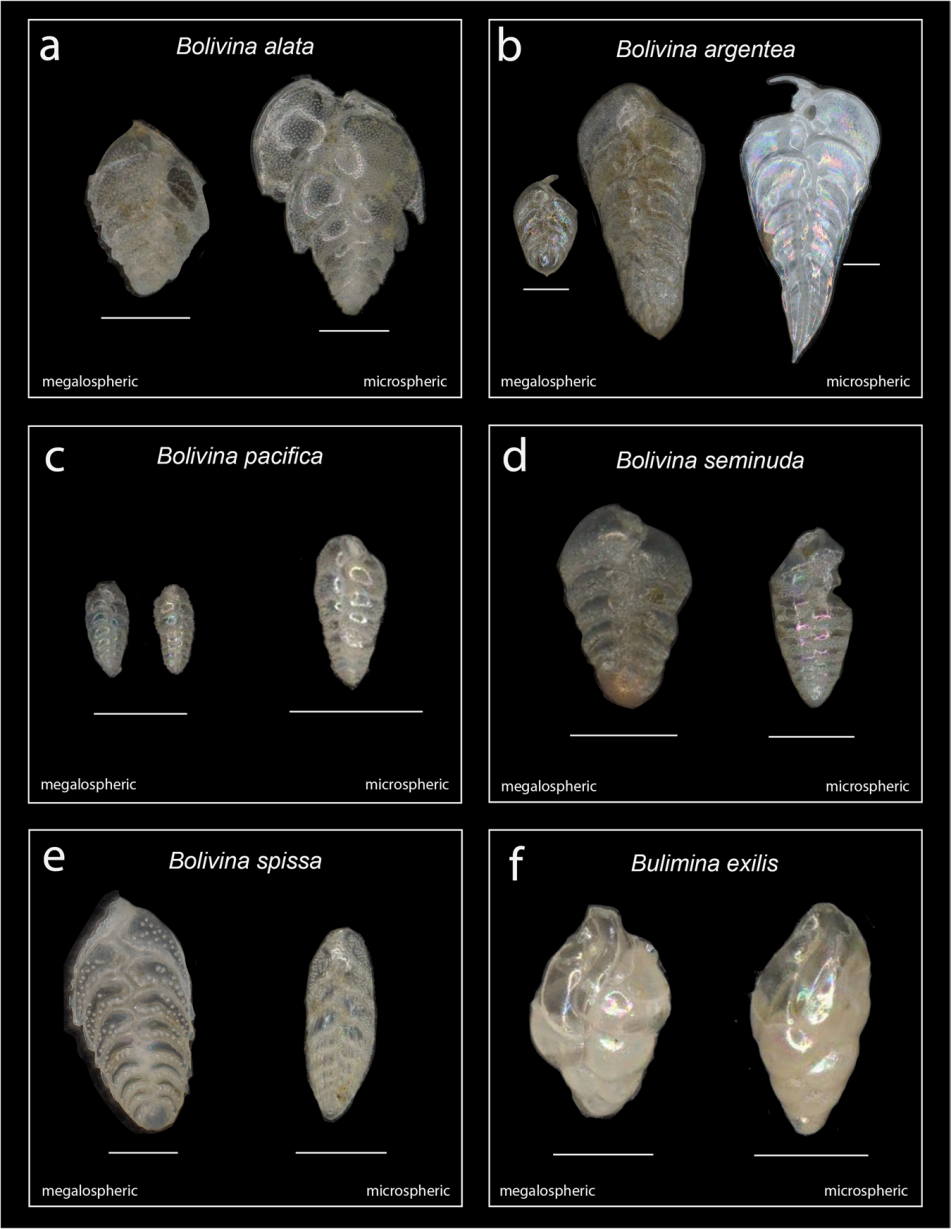
Common biserial benthic foraminifera from site MV1012. (**a**) *Bolivina alata*; (**b**) *B. argentea*; (**c**) *B. pacifica*; (**d**) *B. seminuda*; (**e**) *B. spissa*; (**f**) *Bulimina exilis*. Megalospheric and microspheric morphotypes within each species are denoted; all individuals are arranged with the proloculus (first chamber) facing downwards. Scale bars denote 100 μm.

**Fig. 6:**
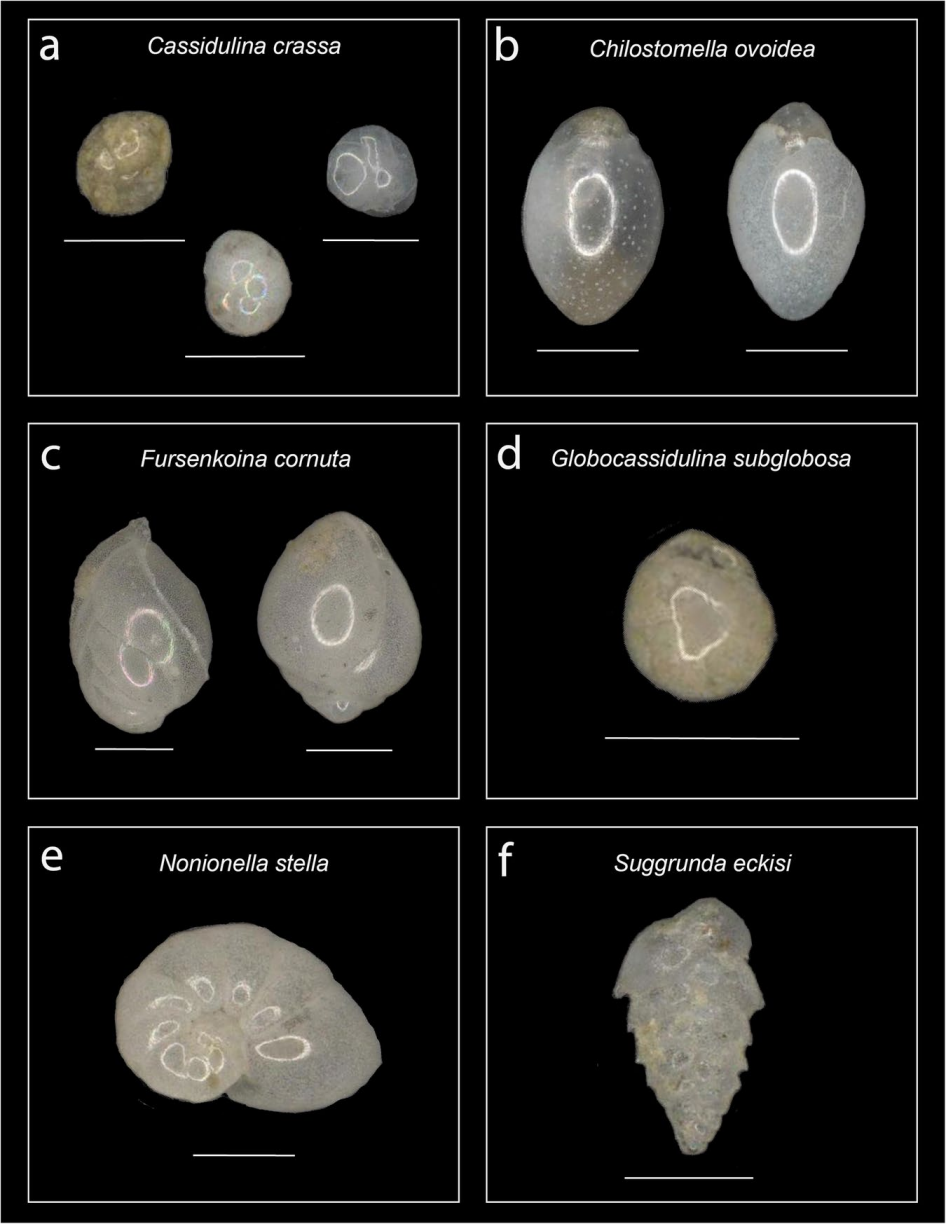
Common benthic foraminifera from site MV1012. (**a**) *Cassidulina crassa*, with individuals show variation in shell coloration; (**b**) *Chilostomella ovoidea*, with individuals showing variation in coloration and porosity; (**c**) *Fursenkoina cornuta*, with individuals rotated ~180° opposite one another; (**d**) *Globocassidulina subglobosa*; (**e**) *Nonionella stella*; and (**f**) Suggrunda eckisi. Scale bars denote 100 μm.

We provide metadata, image, and shape data for all 36,275 objects in the dataset, of which 27,508 are complete and damaged benthic foraminifera from ~60 unique species (Fig. 4), and 26,399 for which shape information was successfully extracted using *AutoMorph*. The tables within this data report provide relevant metadata, summary statistics, and technical validation information. The coring location and an overview of core chronology are shown in Fig. 1, and sample ages and split fractions (i.e., aliquot sizes) are reported in Table 1. The workflow employed for sample preparation, imaging, and processing with *AutoMorph* is shown in Fig. 2. Supplementary Table 1 provides references for taxonomic identifications and common synonyms, and reference images can be found in Figs. 5, 6. All data products of this study are available on Zenodo^43^; this repository contains 8 distinct data types uploaded as distinct files, and includes the following:

(I) *bulk_images.zip*: Bulk images with objects identified by *segment* boxed in red
(II) *individual_images.zip*: EDF images of individual objects within the dataset
(III) *identification_files.zip*: Classifications for individual objects, including both general categories and species-specific classifications (when possible) for benthic foraminifera
(IV) *cleaning_scripts.zip*: Directory containing R scripts used to clean object category misspellings or inconsistencies
(V) *outline_images.zip*: EDF images of objects successfully extracted for 2D outlines and measurements; included for quality control. This includes one text file (unextracted_objects_2D.txt) listing objects with failed extractions
(VI) *2d_coordinates.zip*: CSV files containing all extracted outline coordinates for each of the samples imaged, a text file of failed 2D extractions (unextracted_objects_2D.txt), and a summary CSV file including coordinates for all extracted objects (all_coordinates.csv)
(VII) *2d_properties.zip*: 2D measurements for all objects
(VIII) *metadata_tables.zip*: Tables 1–3 and Supplementary Table 1 from this publication, describing sample metadata, including site coordinates, sample names, object information, and summary statistics

**Table 3:** Technical validation measurements.

Sample Name	Obj. Num.	Species	ImageJ			AutoMorph			Minor Axis Difference	Major Axis Difference	Area Difference
			Minor Axis (µm)	Major Axis (µm)	Area (µm2)	Minor Axis (µm)	Major Axis (µm)	Area (µm2)			
MV1012-BC-3	5	*Globocassidulina subglobossa*	110.5	126.4	10798	112.1	129.5	11369	1.014	1.025	1.053
MV1012-BC-3	7	*Suggrunda eckisi*	96.6	194.9	14173	99	195.2	14764	1.025	1.002	1.042
MV1012-BC-3	14	*Suggrunda eckisi*	98	169.1	11609	95.7	157	11524	0.977	0.928	0.993
MV1012-BC-3	16	*Bolivina pacifica*	73.5	144	7678	75	141.2	8118	1.02	0.981	1.057
MV1012-BC-3	9	*Suggrunda eckisi*	116.6	194.4	16652	115.8	182.8	16315	0.993	0.94	0.98
MV1012-BC-3	4	*Bulimina exilis*	111.5	133.9	10155	109.8	128.9	10986	0.985	0.963	1.082
MV1012-BC-4	20	*Bolivina argentea*	297.4	928.9	207898	320.4	890.8	213158	1.077	0.959	1.025
MV1012-BC-4	53	*Fursenkoina cornuta*	324.9	461.2	107255	328.8	460.8	118560	1.012	0.999	1.105
MV1012-BC-6	158	*Chilostomella oolina*	162.8	259.1	32862	165.1	255.3	33051	1.014	0.985	1.006
								***Mean Diff****.*	1.01	0.98	1.04
(IX) *forams.zip:* Images formatted for use with Tensorflow workflows and associated image labels for object and species identity and fragmentation state (fragment_labels.csv), along with a Tensorflow dataset descriptor (forams.py) and an instructional vignette (README.txt)

## Technical Validation

Technical validation occurred at several steps in the image processing pipeline to ensure that measurements were consistent across samples, and that all measurements were extracted from outlines that were true to the original sample shape. The major validation steps occurred at the object selection, shape extraction and size measurement, and object classification phases.

### Object selection

The *AutoMorph* segment module produces a bulk image overview that provides an object number for each individual segmented object, which is boxed in red for ease of identification (Fig. 2; full sample set of boxed images available in data citation). Full-sample images taken on the Keyence VHX-7000 digital imaging microscope were output as extended-depth-of-focus (EDF) images, and these EDF images were passed to the segment module in ‘sample’ mode to produce a series of boxed images, which denoted the object numbers for each segmented individual, for visual validation prior to finalizing the segmentation output. Each boxed image was visually checked to verify that most, if not all, microfossils were identified and segmented from each image. If this visual check failed—i.e., some or many microfossils were excluded from the segmentation—image selection parameters were adjusted in segment to optimize segmentation. Once an optimal parameter was identified, the segment module was run in ‘final’ mode to create individual images of each of the objects identified from the full-sample image. These individual images provide the basis for the *run2dmorph* module, which produces 2D measurements, and for taxonomic identification.

### Shape extraction

2D shape extraction occurred via the *AutoMorph* run2dmorph module, which takes as input individual 2D EDF images and produces outline coordinates, measurements, and validation images with outlines overlain atop the input image. The quality of 2D shape extraction was checked visually for the first 100 objects in a slide using these outline-object overlays. run2dmorph also outputs a list of objects with failed outline extractions for each sample processed; these are provided alongside 2D shape data in the Data Citation. When a majority of complete benthic foraminifera failed to extract, the run2dmoprh routine was re-run with adjusted image extraction parameters to attain the best possible extraction.

Successful shape extractions can sometimes produce outlines that do not reflect the true outline of the specimen. To account for these errors, outline-based measurements can be filtered by using a rugosity threshold. Because the threshold of filtering needed may vary based on the data application, we provide all outline-based measurements in the Data Citation. See **Usage Notes** for suggested filtering thresholds.

### Size measurements

The accuracy of 2D size extraction was confirmed by measuring individuals with successful shape extraction on a Keyence VHX-7000 imaging microscope. Table 3 contains 10 benthic foraminifera from 7 species used to check 2D size extraction. Individuals were measured along their major and minor axes and outline-based area measurements were collected using ImageJ measurement software. We find that automated and “human” measurements are comparable, such that *AutoMorph* measurements range, on average, from 97% to 104% of hand measurements. Average differences between major and minor axes was ~5 μm. Extended technical evaluations of *AutoMorph* measurements can be found in publications associated with the software suite^2–4^.

### Object classification

6 individual researchers worked simultaneously to classify objects from images. These researchers were undergraduate students without prior knowledge of foraminiferal morphology or taxonomy. In order to ensure inter-identifier consistency, all were trained to identify objects using a set of samples pre-identified by the first author (SSK), building on object categories outlined in Elder *et al*.^4^. Each sample was not considered completely identified until at least two unique identifiers provided classifications for all objects within the sample. These object classifications were then compared, and disagreements between identifiers were checked by SSK, who provided the final classification. Additionally, all identifiers provided a confidence (scale of 1–3, from least to most confident) for each object classification, which allowed for identifications with low confidence to be checked and updated. In cases where objects remained difficult to identify with certainty, the object classification was changed to ‘unknown’ to prevent misidentification. Errors are described briefly here, with each classification category described in more detail in **Usage Notes**. Most misidentifications were for species-level taxonomic classifications of benthic foraminifera (see below). For general object classification, classification errors included misidentification of non-benthic objects, including radiolaria, planktonic foraminifera, diatoms, and pteropods, which, when classified in error, were typically identified as ‘benthic foraminifer fragments’ or ‘junk.’ Images that contained multiple objects (e.g., had not been properly arranged during the sample preparation step and as a result were touching, or had overlapping outlines) were also misclassified when individual classifiers chose to identify one or more objects rather than classify them as ‘touching’. Chunks of consolidated sediment were typically poorly classified, and as a result, were assigned to the ‘unknown’ category. In cases where individual images were of large individuals, the segmented image boundary occasionally contained other, smaller individuals (which typically were captured within their own segmented images), some of which were erroneously classified alongside the larger individual. To remedy inconsistent object classifications, visual checks (as described above) were used to reassign object categories. Following visual checks, automated cleaning scripts were employed to remedy misspellings or inconsistent spellings among object categories. These scripts are included within Data Citation 1.

### Taxonomic classification

Taxonomic classification of benthic foraminifera occurred during the same classification step as general object classification (see Table S1.1 for taxonomic references). Benthic foraminifera were identified to species whenever possible, and identifiers were trained to make species-level identifications using a set of reference images classified by SSK. Reference images for twelve of the most common species can be found in Figs. 5, 6. During object classification, identifiers classified benthic foraminifera to species and provided a confidence level for their classification. While the majority of confident classifications were for benthic foraminifera with complete or partially-damaged shells, on occasion classifications could be made from fragmentary pieces of shell (see **Usage Notes** for suggestions on how to filter out these specimens when using morphometrics data). In total, ~60 unique species were identifiable from all samples, and are listed in Supplementary Table 1.

## Usage Notes

Following their collection and preparation, many of the samples used in this study were picked for fish otoliths and plastic particles prior to the present study. However, the remainder of objects, including the benthic foraminifera on which we focus, were, to our knowledge, unbiased by previous research efforts undertaken on this material. It is worth noting that the benthic foraminifera that we observe for this study represent death assemblages, and as such may not be fully representative of the composition of living communities at the time of sediment layer deposition.

### Time averaging

In studies of nearby sites in the Southern California Borderlands, death assemblages of benthic foraminifera are shown to differ in species composition, proportion, and distribution when compared to living assemblages^44^. However, these studies have lower temporal resolution than the present contribution, and may be observing time-averaged differences in assemblages that result from changes to shelf, slope, and basin environments that have taken place over the last several hundred years^45^. Yet even within the well-resolved sediments of the Santa Barbara Basin, there may be migration of benthic foraminifera between sediment layers. Some species undertake daily to seasonal migrations between the sediment-water interface and the uppermost centimeters of sediment^46,47^, and as a result, sediment layers of a given age may contain individuals from younger populations. Because vertical migration may be less pronounced during periods of anoxia^46^, the dysoxic waters of the Santa Barbara Basin may serve to limit this effect. Although the effects of time-averaging and differential vertical migratory behavior are likely small for the Santa Barbara Basin, mere differences in population dynamics between taxa can also produce live and death assemblage discrepancies. Populations with shorter longevity will contribute tests to the death assemblage at a higher rate than long-lived ones, and will thus be relatively overrepresented in the fossil record. We caution that any analyses that utilize these data take benthic foraminifer ecology, population dynamics and the broader environmental and temporal setting of these samples into account.

### Sample preparation and classification

While the 63 μm size limit we apply here can be considered a general lower bound for the size of objects within our samples, some smaller particles may have been retained during processing stage. These smaller objects should not be considered representative of the <63 μm fraction and should be excluded from the majority of data applications. In addition, while objects other than benthic foraminifera are included within these data, the majority were not intentionally picked out of the larger sample and should not be considered representative. However, a few classes were picked within intentionality and can be considered a representative fraction, where the entirety of the sample has been picked for a given class. These include fish teeth, shark dermal denticles, pteropods, and other small (including larval) bivalve and gastropod shells. These, alongside the benthic foraminifera, are the only objects that should be considered for future systematic and ecological studies that employ these data. Each object was classified by a human observer (i.e., identifier) and placed into one of 15 categories along with an indication of confidence in the classification (1: not confident, 2: somewhat confident, 3: very confident).

Broad classification categories are defined following Elder *et al*.^4^. ‘Junk’ denotes any fibers, inorganic crystalline structures, sand, rocks, captured images of light reflecting off of the background imaging plate, and other unidentifiable, non-biological forms. ‘Planktic’ indicates any planktonic foraminifer, and includes shells that are complete, damaged, and fragmented. ‘Fragment’ includes any fragment of a benthic foraminifer that is not easily identifiable to species. ‘Gastropod’ denotes any gastropod shell, other than pteropods. ‘Pteropod’ denotes any pteropod shell, and includes shells that are complete, damaged, and fragmented. ‘Bivalve’ denotes small (potentially larval) bivalve shells. ‘Fish tooth’ denotes any fish dental structure, but does not include shark dermal denticles. ‘Dermal Denticle’ denotes any shark dermal denticle of any species. The ‘Radiolaria’ category contains radiolarians, ‘Diatom’ contains diatom frustules, ‘Echinoid’ contains echinoid fragments (including spines), ‘Spicule’ contains sponge spicules, and ‘Ostracod’ contains ostracods. In each of these categories, complete or larger individuals in clear, well-focused images were typically identified with greater confidence than broken or smaller objects, or those in out-of-focus images. Finally, ‘Touching’ denotes any images of multiple objects, which cannot be given a single identification. Objects in direct or very near contact are unable to be used for accurate 2D size and shape extraction, and should be excluded from any morphometric analyses.

### Morphometrics and machine learning

Morphometric data should be checked prior to analyses according to the given use case. For example, data used for a study of body size may be filtered to remove poorly-extracted outlines by applying a rugosity filter, where objects with a rugosity greater than a given threshold are excluded from analyses. Other morphometric outputs that can aid in automated cleaning include aspect ratio and the outline coordinates.

Objects for which 2D size and shape extraction failed are listed in each relevant measurement file. Metadata including pixel sizes used for automated measurement can be found in the labels attached to each bulk and individual image provided in the data file *individual_images.zip* of Data Citation 1. These pixel sizes can be used for future measurement via *AutoMorph* or other morphometric software. Additional metadata provided via image labels includes the sample name, object number, age, locality name, where images were processed, and the identity of the individual who processed the images. This metadata is permanently associated with images to ensure that no information is lost should these images be separated from other data files.

Select samples were run with an incorrect size factor. As a result, the morphometric measurements for these samples were corrected in post-processing using a set of conversion factors. Table 4 reports these samples and the conversion factors for linear and 2D measurements. Note that while measurements for these samples have been corrected, the scale bars will not provide an accurate measurement of pixel size (i.e., μm/pixel); corrected pixel sizes are reported in Table 4.

**Table 4:** Pixel size conversion factors.

Sample	Corrected Pixel Scale (µm/pixel)	Linear Conversion Factor	2D Conversion Factor
MV1012-KC1-1-25	1.23	1.52	2.31
MV1012-KC1-1-52	1.25	1.56	2.44
MV1012-KC1-1-64	2.38	5.67	32.11
MV1012-KC1-1-103	1.2	1.45	2.09
MV1012-KC1-1-117	1.23	1.52	2.31
MV1012-KC1-1-124	2.38	5.67	32.11
MV1012-KC1-2-9	1.2	1.45	2.09
MV1012-KC1-2-28	1.2	1.45	2.09
MV1012-KC1-2-49	1.23	1.52	2.31
MV1012-KC1-2-59	0.64	0.4	0.16
MV1012-KC1-2-64	2.38	5.67	32.11
MV1012-KC1-2-68	1.2	1.45	2.09
MV1012-KC1-2-80	1.27	1.61	2.58
MV1012-KC1-2-87	1.23	1.52	2.31
MV1012-KC1-2-103	1.25	1.56	2.44

Images in the Tensorflow dataset have been pre-processed for machine learning workflows and can be readily loaded into python with two lines of code (Data Records for readme document). Machine learning models and outputs should be used with caution in studies where quantification of the abundance of rare species is a central goal, as the models we show here will require further tuning and optimization to accurately report rare species identity. Fragmentation state labels should not be considered representative without information on species identity, as all debris and other non-microfossil objects are labeled as ‘unbroken’ by default Fig. 3.

## Data Availability

Images were processed using *AutoMorph* software, which is described in Hsiang *et al*.^2^ and freely available on GitHub at www.github.com/HullLab/AutoMorph. The classifier software used to tag images with taxonomic identifications can be found on GitHub at https://github.com/GregDMeyer/classifier.
